# Numerical Investigation on Precipitation Hardening of Mg-Gd Alloys

**DOI:** 10.3390/ma17061393

**Published:** 2024-03-19

**Authors:** Yulong Ge, Chunyan Yang, Yuwei Ma, Yang Chen, Manoj Gupta

**Affiliations:** 1 Department of Mechanical and Electrical Engineering, Guilin University of Electronic Technology, Guilin 541004, China; 18369107108@163.com (Y.G.); 21012302114@mails.guet.edu.cn (C.Y.); 2 Department of Architecture and Transportation Engineering, Guilin University of Electronic Technology, Guilin 541004, China; myw@guet.edu.cn; 3 Department of Mechanical Engineering, National University of Singapore, 9 Engineering Drive 1, Singapore 117575, Singapore

**Keywords:** Mg alloys, second-phase particles, precipitation hardening, finite element model, interface strength

## Abstract

The second-phase particles in magnesium alloys could affect the mechanical properties of the material significantly. In this work, 3D finite element models with explicit incorporation of second-phase particles are established. The simulations are calibrated with the experimental results of the Mg-1Gd alloy. The influences of factors, such as the particle distribution, size, and orientation of cylindrical particles, on precipitation hardening are investigated in detail. Three interface conditions between particles and the matrix—perfect bonding and high- and low-strength bonding—are studied at the same time. The interface conditions are shown to exert a stronger influence on precipitation hardening compared to the factors of particle distribution and size. In contrast, the influence of the orientation of cylindrical particles at grain boundaries outweighs the effect of interface property. When second-phase particles are relatively large and all located at grain boundaries, the hardening effect can be improved, and the magnesium alloy shows relatively high flow stress. However, the high hardening effect from the second-phase particles could result in high local stress concentration and possible early failure or low ductility of Mg alloys.

## 1. Introduction

Magnesium alloys stand as the most lightweight structural metallic materials to date. The advantages of high specific strength and specific stiffness endow magnesium (Mg) alloys with promising potential applications in the industries of aerospace, automotive, and consumer electronics [1,2,3,4]. Nevertheless, due to the relatively low strength and poor ductility of Mg alloys, the industrial applications of Mg alloys are restricted [5]. In recent years, many research efforts have been made to control the microstructure of magnesium alloys to improve their mechanical properties, such as through judicious alloy design [6,7] and the addition of particles into the Mg matrix [8,9]. Specifically, rare earth elements commonly show a strong influence on the mechanical responses of Mg alloys [10,11,12]. Second-phase particles (SPPs) with rare earth elements in the matrix are reported to yield Mg alloys with enhanced mechanical properties (e.g., yield strength, ultimate strength, and ductility) [13,14].

The rare earth element Gadolinium (Gd), with a notable solid solubility in Mg, has an outstanding precipitation-strengthening effect in Mg alloys [15,16]. Gd stands out as a prominent alloying element to improve the strength and ductility of magnesium alloys. Owing to grain boundary segregation, the SPP in the alloys (e.g., Gd-rich second phases in Mg-Gd alloys) are usually formed along the grain boundaries or at the triple grain boundary junctions, and only a few particles are located in the grain interior [17,18,19]. In general, the presence of second-phase clusters or particles decorating the grain boundaries tends to induce local stress concentration, and, consequently, it leads to a reduction in the ductility of alloys. The nonuniform distribution of particles is commonly considered an adverse factor for mechanical properties [17,20,21]. However, in recent years, several studies [22,23,24,25,26] have reported enhanced mechanical performance in composite materials with different configurations through controlled distributions of SPPs, which surpassed the properties of composites with uniformly dispersed particles. It is worth noting that factors such as shape, size, volume fraction, distribution, and the interfacial properties between particles and the matrix could influence the mechanical properties of Mg alloys significantly [8,27]. Nowadays, it is still a challenge to obtain a comprehensive understanding of the influence of SPPs on the mechanical properties of Mg-Gd alloys.

Actually, it is difficult to quantitatively analyze the influence of individual factors related to SPPs mentioned above through experimental studies. Therefore, establishing a reliable simulation model is an effective and economical method to study the mechanical properties of Mg alloys. Also, using such a developed simulation model, it would be possible to obtain some useful data that are difficult to obtain through experiments. Some researchers [7,21] have employed theoretical models to estimate the contributions of various factors (e.g., the shape and location of second-phase particles), thereby predicting the material’s mechanical properties. Nevertheless, due to the complexity of the issue, theoretical analyses still exhibit certain limitations. As a result, finite element simulation, as an alternative technique, has been widely applied to predict the mechanical properties and failure mechanisms of particle-reinforced composites [22,23,24,25,28,29]. Weng et al. [30] established a model with randomly distributed particles in the Al matrix materials to investigate the influence of particle morphology, size, and interfacial damage on the mechanical properties of composite materials. Gao et al. [23] established a three-dimensional model characterized by a network distribution of particles and, subsequently, they assessed the impact of this network structure on the strength of the material as well as deformation behavior, including fracture.

Material properties of the SPP can be calculated through nanoindentation techniques, while the distribution and morphology of the SPP can be acquired using X-ray Computed Tomography (XCT) imaging techniques. Sarvesha et al. [31] reported the influence of SPPs with authentic microstructures on the deformation behavior of AZ80 alloys. Tian et al. [32] quantified the geometrical parameters of SPPs using XCT and investigated the impact of SPPs with multiscale microstructures on the deformation response of ZK60 alloys. Dastgerdi et al. [33] studied the influence of particle cluster size, localized volume fraction within clusters, and particle agglomeration for Mg alloys. These studies indicate that the deformation behavior of Mg alloys is closely related to the microstructure, distribution, and interface damage of the SPP. However, the exact influence of SPPs on the overall mechanical performance of Mg alloys remains unclear, underscoring the necessity of further investigation on the impact of SPPs on the mechanical response of Mg alloys.

Precipitation hardening (or hardening by SPPs) means that due to the presence of precipitations or SPPs, the flow strength and/or the yield strength of the metallic materials could be improved [34]. In the present work, the influence of SPPs on mechanical properties of Mg alloys with Gd is investigated employing finite elements models with explicit incorporation of particles in the matrix. The experimental results of the Mg alloy Mg-1Gd are adopted for comparison. The influences of the particle distribution, particle size, and orientation of cylindrical particles coupled with varied interface strengths between the SPP and the matrix are studied in detail.

## 2. Experiments

### 2.1. Material Preparation and Microstructural Characterization

Cast ingots of pure Mg and Mg-1.0 wt.% Gd alloys were produced by a disintegrated melt deposition (DMD) technique [35] using pure Mg (>99.9% purity) and Mg-30.0 wt.% Gd. Subsequently, with an extrusion ratio of 20.25:1, the material was hot-extruded into rods with a diameter of 8 mm at 300 °C.

Microstructures of the specimens were observed using an optical microscope (Leica DM2700M, Leica Microsystems Ltd., Wetzlar, Germany). An Abrams Three-Circle Procedure (ASTM E112) was used to measure the grain sizes of the two materials using LAS X software (version 5.0) from the OM images. The samples were ground and then polished by 0.5 μm diamond suspension to a mirror finish surface. The etchant solution comprised 1 g oxalic acid, 1 mL nitric acid, 1 mL acetic acid, and 150 mL distilled water, and the holding time for etching was in the range of 20–30 s.

The microstructural characterizations of Mg-Gd alloys were performed using a Field emission Scanning Electron Microscope (FE-SEM) (Gemini SEM 300, Zeiss Group, Oberkochen, Germany) equipped with an energy dispersive spectroscopy (EDS) detector with an acceleration voltage of 20 kV and a working distance of 10 mm. The volume fraction and size of the SPP were measured using the image analysis software ImageJ (version 1.54d) of ten FE-SEM images. At least 200 particles were analyzed per image to obtain the average value.

### 2.2. Mechanical Properties

Nanoindentation was employed to characterize the second-phase particle using Bruker’s Hysitron nanoindenters (Hysitron TI 950, Bruker corp., Eden Prairie, MN, USA). A diamond Berkovich indenter tip was employed with a constant loading and unloading rate of 0.5 mN/s and a dwell time of 2 s. Mg-Gd precipitations in the samples were subjected to a peak load of 2.5 mN. At least five indentation tests were performed to obtain an average value.

Quasi-static uniaxial tensile tests were performed using dog bone tensile specimens (a gauge length of 25 mm and a gauge diameter of 5 mm) at room temperature with a constant strain rate of 1 × 10^−3^ s^−1^ using the electronic universal testing machine (CMT5105, SANS Ltd, Shenzhen, China). During the tests, three-dimensional digital image correlation (DIC) technology (XTOP XTDIC-CONST-HS) was employed to measure the axial deformation. Three specimens were tested for each material.

## 3. Finite Element Modeling

### 3.1. Microstructural Modeling

The distribution of SPPs could affect the mechanical properties of Mg alloys significantly [36]. In this work, 3D representative volume element (RVE) models with varied distributions of SPPs are built, including one with randomly distributed SPPs, one with all SPPs located at grain boundaries, and one with half of the SPPs at grain boundaries and half of the SPPs in grain interiors. The distributions are controlled using the random sequential adsorption (RSA) method. To identify the location of grain boundaries, the open resource software Neper (version 4.8) [37] is employed to generate a polycrystalline model with a similar grain size to that of the Mg-1Gd alloy, and, subsequently, locations of the grain boundary are extracted from this polycrystal model. The shape, size, and volume fraction of the particles are determined based on experimental results.

**Figure 1:**
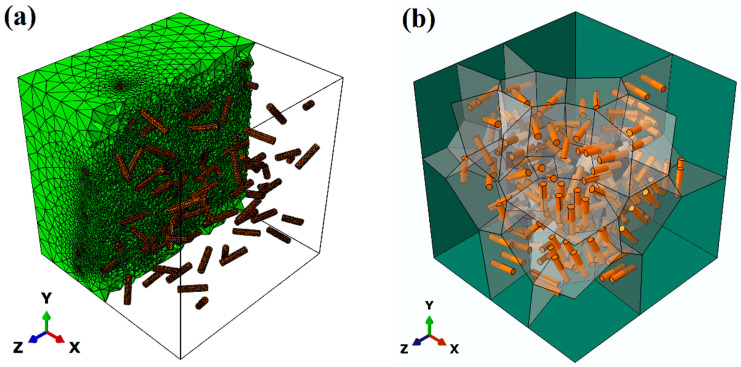
(**a**) A 3D RVE model with the distributed SPPs; (**b**) a geometrical model with all the SPPs located at the grain boundaries (gray planes denote the grain boundaries).

The 3D finite element (FE) models, as shown in Figure 1a, are employed to conduct numerical simulations using ABAQUS/Standard [38]. The size of the 3D RVE model is 10.0 μm × 10.0 μm × 10.0 μm. A 4-node linear tetrahedron element (C3D4) [39] is used to mesh the 3D models of the matrix and particles. Zero-thickness cohesive 6-node elements (COH3D6) [39] are inserted between the particles and the matrix to represent interfacial bonding. The meshes of the model are refined to ensure numerical accuracy. As an example, Figure 1b shows one 3D microstructural model with all the SPPs in the Mg-Gd alloy located at the grain boundaries, which are indicated by gray planes.

### 3.2. Material Properties

During deformation, the matrix and particles are considered to behave as elastic-plastic and linear elastic materials, respectively. For the elastic properties of the particles, Young’s modulus and hardness are set at 57.8 ± 4.79 GPa and 4.11 ± 0.30 GPa, respectively, based on reported experimental results [40]. The mechanical properties of the matrix are determined by reverse analysis from load–displacement curves obtained by nanoindentation tests in this study.

**Figure 2:**
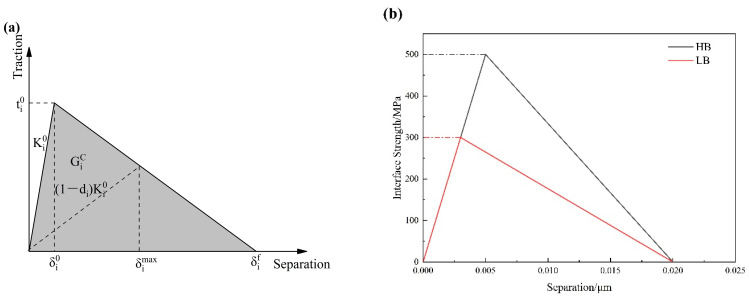
(**a**) Schematic diagram of a bilinear traction–separation law [30]; (**b**) the bilinear cohesive model with parameters for interfaces HB and LB.

The constitutive behavior of the cohesive elements is modeled using a bilinear traction–separation law, as shown in Figure 2a. The quadratic nominal stress criterion is used to describe the damage initiation of the cohesive zone model (CZM) [41] as follows:

(1) $${\left\{\frac{\langle t_{n}\rangle }{t_{n}^{0}}\right\}}^{2}+{\left\{\frac{\langle t_{s}\rangle }{t_{s}^{0}}\right\}}^{2}+{\left\{\frac{\langle t_{t}\rangle }{t_{t}^{0}}\right\}}^{2}=1$$

where $t_{i}^{0}$ (*i = n, s, t*) represents the peak traction in the normal and two shear directions, respectively. The symbol ⟨⟩ denotes the Macauley bracket [42] defined as ⟨R⟩ = R when R ≥ 0 and ⟨R⟩ = 0 when R < 0, indicating that pure compression does not lead to damage.

A fracture criterion based on a power law formulation is employed to describe the evolution of damage with the following expression [39]:

(2) $${\left\{\frac{G_{n}}{G_{n}^{C}}\right\}}^{\alpha }+{\left\{\frac{G_{s}}{G_{s}^{C}}\right\}}^{\alpha }+{\left\{\frac{G_{t}}{G_{t}^{C}}\right\}}^{\alpha }=1$$

where $G_{i}$ (*i = n, s, t*) are fracture energies in the normal and two shear directions, respectively. $G_{i}^{C}$ refers to the critical fracture energies required to cause failure. α is the power index, and the value is set to be 1 based on Ref. [30].

The bilinear cohesive law is governed by three independent parameters: interface strength (*t*), initial stiffness (K), and fracture energy ($G_{i}^{C}$) (or failure displacement ($\delta_{i}^{f}$)). Three interface conditions are proposed to be studied in this work: perfect interface bonding (PB), interface bonding with high strength (HB), and interface bonding with low strength (LB). The interface stiffness could affect the elastic behavior of the material. It is determined by calibrating the simulated stress–strain curve with respect to the experimental results (a detailed explanation is presented in Section 3.4), and the value is set to be 100 GPa for all three interface conditions. Therefore, when no damage occurs, the macroscopic responses of the three interface scenarios are nearly identical in the elastic regime. The interface strength in the normal and shear directions is set to be equal. The parameters for the interface are listed in Table 1. The parameter for the HB interface is obtained by fitting the stress–strain curves obtained from uniaxial tensile experiments using finite element simulations. The two interface conditions, HB and LB, as described by the bilinear cohesive model, are shown in Figure 2b.

### 3.3. Periodic Boundary Conditions

**Figure 3:**
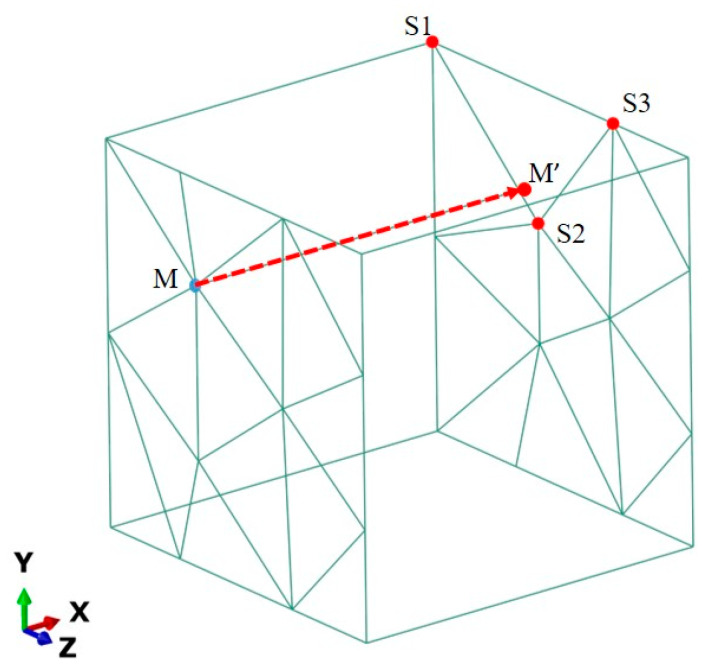
Schematic diagram of periodic boundary conditions.

In order to make the RVE model represent the mechanical behavior of bulk materials well, periodical boundary conditions are applied to the RVE faces. A schematic diagram illustrating the periodic boundary conditions is shown in Figure 3.

The boundary conditions can be expressed as follows [43]:

(3) $$u_{i}^{j+}\left(M\prime \right)=[\begin{array}{ccc} N_{1} & N_{2} & N_{3}]\cdot \left[\begin{array}{c} u_{i}^{j-}\left(S_{1}\right)\\ u_{i}^{j-}\left(S_{2}\right)\\ u_{i}^{j-}\left(S_{3}\right) \end{array}\right] \end{array}+\bar{\varepsilon_{ik}}\cdot L(i,j=1,2,3)$$

where $\bar{\varepsilon_{ik}}$ is the unit cell average strain and $u_{i}$ are the displacements of the node in the x, y, and z directions, respectively. The superscripts *j*+ and *j*− indicate the positive and negative directions along the axis, respectively. *L* is the original length of the cubic RVE model. *M* is any node on the main plane, and M′ is the node projected from *M* to its slave plane, as shown in Figure 3. In the figure, the triangle unit surrounding the corresponding point M′ on the slave plane is $∆S_{1}S_{2}S_{3}$. [*N*] is the element shape function matrix corresponding to point *M* on the surface. A displacement load is applied in the x direction so that the maximum tensile strain reaches 10%. The numerical true stress–strain curves in the tensile direction of the composite in the unit cell are calculated by reaction force and displacement on the surface X.

### 3.4. Model Validation

**Figure 4:**
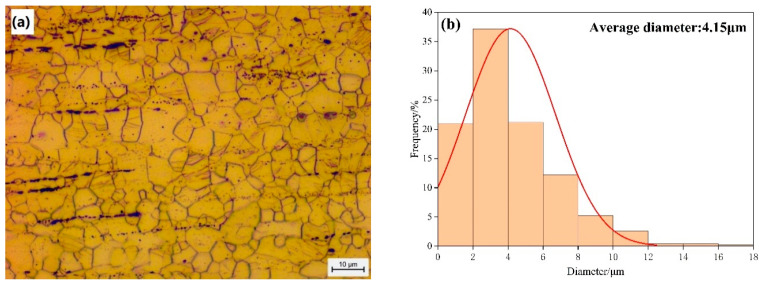
(**a**) Micrograph showing grain characteristics of the Mg alloy Mg-1Gd; (**b**) statistical distributions of the grain size in the Mg alloy Mg-1Gd.

**Figure 5:**
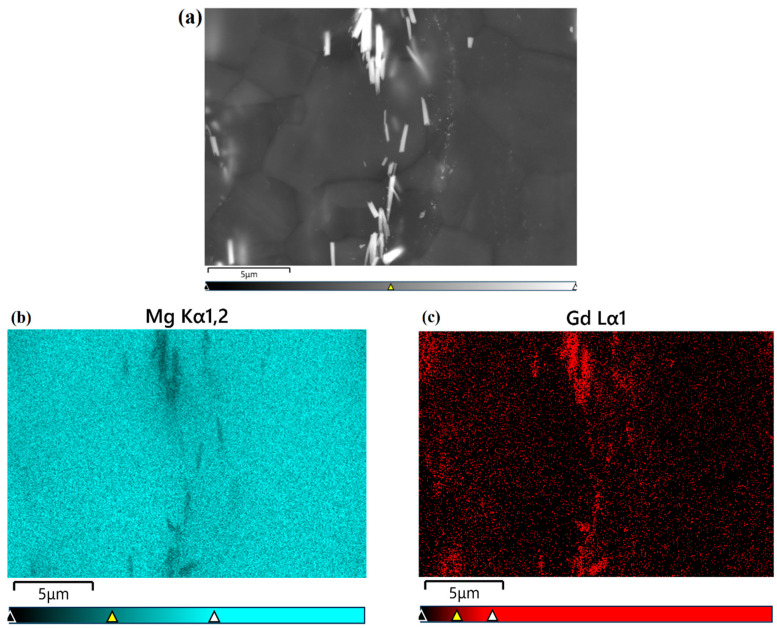
(**a**) BSE image of the Mg alloy Mg-1Gd; (**b**) EDS map of Mg in the alloy Mg-1Gd; and (**c**) EDS map of Gd in the alloy Mg-1Gd.

Figure 4a shows the typical image of grain morphology for the Mg alloy Mg-1Gd from optical microscopy. The statistical distribution of grain size is given in Figure 4b, and the average grain size is 4.15 μm. Figure 5a shows the microstructure of the alloy Mg-1Gd using backscattered electrons (BSEs) in FESEM, and the corresponding EDS maps of elements Mg and Gd are given in Figure 5b,c, respectively. The BSE image and EDS maps confirm the presence of Gd-rich second-phase particles, which are noted by white rods precipitated along the grain boundaries in the matrix. Quantitative analysis was performed to assess the volume fraction, maximum Feret diameter (defined as the maximum distance between two parallel tangents to the SPP), and aspect ratio (defined as the ratio of the maximum to minimum Feret diameter) of the second phase, and the results are shown in Table 2.

**Figure 6:**
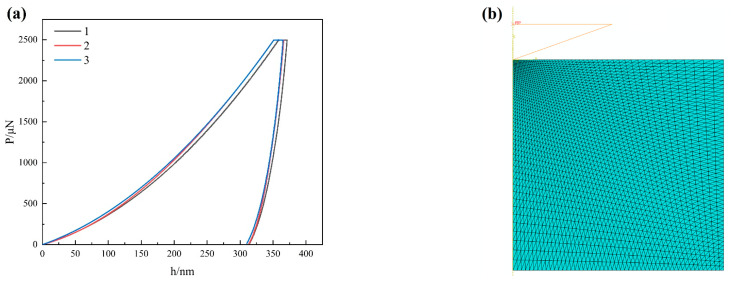
(**a**) Load–displacement curves for the matrix material by nanoindentation tests; (**b**) 2D axisymmetric FE model of nanoindentation tests.

The load–displacement curve obtained from nanoindentation tests on the matrix of the alloy Mg-1Gd is shown in Figure 6a. The material properties of the matrix and SPP are listed in Table 3. In this table, the elastoplastic properties of α-Mg are estimated by the following equation [44]:

(4) $$\frac{1}{E_{r}}=\frac{1-\vartheta^{2}}{E}+\frac{1-\vartheta_{i}^{2}}{E_{i}}$$

where $E_{r}$ is reduced Young’s modulus. *E* and ϑ are Young’s modulus and Poisson’s ratio for the matrix, respectively. The value of ϑ is fixed at 0.3. $E_{i}$ and $\vartheta_{i}$ are Young’s modulus and Poisson’s ratio for the indenter, and their values are 1140 GPa and 0.07, respectively.

**Figure 7:**
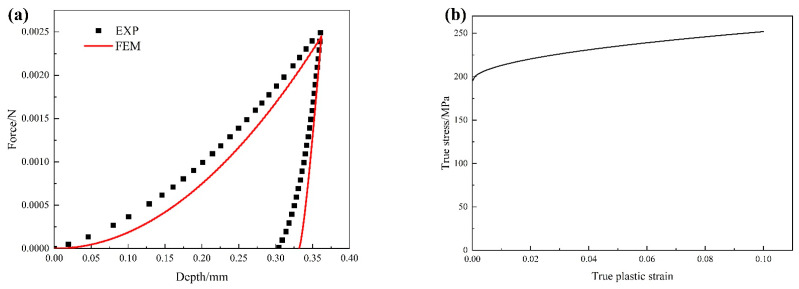
(**a**) Comparison of load–displacement curves from both experiment and FE simulation; (**b**) the simulated plastic behavior of the matrix based on calibrated material parameters.

To describe the plastic behavior of the matrix, a two-dimensional axisymmetric finite element model (see Figure 6b) is built for reverse analysis [45], which refers to calculating the elastic–plastic properties of the matrix from the given indentation data. The standard Berkovich indenter [45] used in experiments is equivalently modeled as a rigid conical indenter with the same projected area (half cone angle of 70.3°). The Ludwick hardening model is applied to characterize the plastic behavior of the matrix material during quasi-static uniaxial deformation [46].

(5) $$\sigma =\sigma_{y}+K{\varepsilon_{p}}^{n}$$

where $\sigma_{y}$ represents the yield stress, *n* is the strain hardening exponent, *K* is the strain hardening parameter, and $\varepsilon_{p}$ is the plastic strain. By adjusting the parameters of $\sigma_{y}$, *n*, and *K*, the resulting load–displacement curve obtained from finite element simulation agrees well with experimental results (as shown in Figure 7a). The values of $\sigma_{y}$, *K*, and *n* are set to be 195 MPa, 180 MPa, and 0.5, respectively. The resultant plastic stress–strain curve of the matrix is illustrated in Figure 7b. 

**Figure 8:**
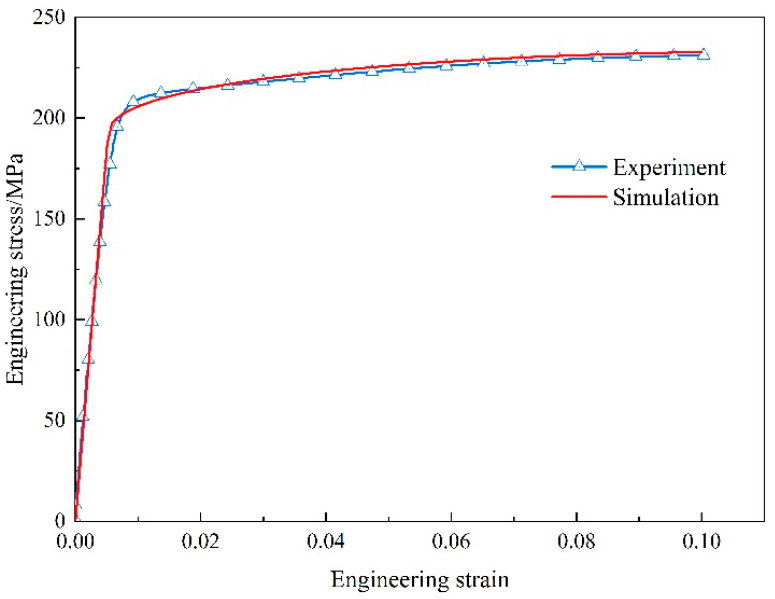
Comparison of the tensile stress–strain curves of the alloy Mg-Gd between experiment and simulation.

As mentioned above, the parameters presented in Table 1, Table 2 and Table 3 are used as input for the model, and an FE simulation model with uniformly distributed second-phase particles (cylindrical particles with random orientations) is constructed. The periodic boundary conditions described in Section 3.3 are applied to the FE model. The interface properties between particles and the matrix significantly impact the material’s performance. However, it is difficult to determine the interface properties by experiment. In response, some researchers investigate interface properties through methods such as first-principle calculations, molecular dynamics simulations, and microscopic FE models [47,48,49,50,51,52]. For different Mg alloys, there is significant variation in the interface properties between the matrix and SPP. Hence, due to the absence of pertinent research data on the matrix/secondary phase interface, this study determined the interface properties by fitting finite element simulations to experimental results. The interface stiffness is set as 100 GPa, with an interface strength of $t_{i}^{0}$ = 500 MPa and an interface fracture energy of $G_{i}^{C}$ = 5 J/m^2^. To validate the reliability of the model parameters, a comparison between the stress–strain curves predicted by FE simulations and experimental results is conducted, as shown in Figure 8. The results indicate that the prediction is in good agreement with the experimental result. This suggests that the parameters adopted for the FE model are effective and will be used for subsequent simulations.

## 4. Results and Discussions

### 4.1. Effects of Particle Distribution and Interface Strength

**Figure 9:**
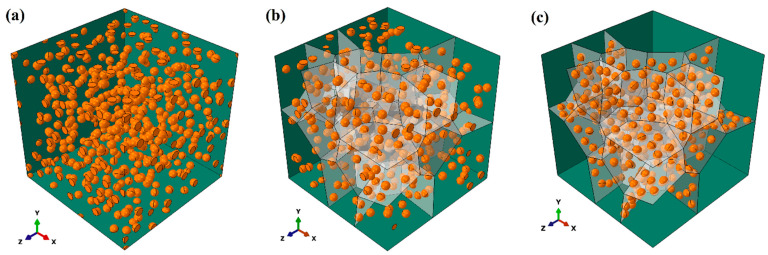
Geometrical models with varied particle distributions: (**a**) homogeneous referred to as D-HO, (**b**) intragranular boundary referred to as D-IB, (**c**) grain boundary distribution referred to as D-GB (gray planes denote the location of grain boundaries).

The distribution of the SPPs within the matrix and interface properties between particles and the matrix would exert strong influences on the effect of precipitation hardening. To investigate the individual and coupled influences from the two factors, three distributions of SPPs in the matrix are considered, and the corresponding FE models are built. Figure 9a shows the homogeneous distribution of SPPs, denoted by D-HO. The particles are uniformly dispersed in the matrix. Figure 9b is the intragranular boundary distribution, denoted by D-IB. In the figure, half of the particles are located along the grain boundaries, and the other half are distributed within the grains. Figure 9c shows the grain boundary distribution, denoted by D-GB. All the particles are distributed along the grain boundaries in this arrangement. For each distribution, three interface conditions are constructed, respectively: perfect interface bonding (PB), interface bonding with high strength (HB), and interface bonding with low strength (LB), which are introduced in Section 3.2. To concentrate on the aforementioned two factors in this part, only spherical particles are considered in the FE models with the same volume fraction of 4%, and the ratio of grain size to particle is set to 7:1.

**Figure 10:**
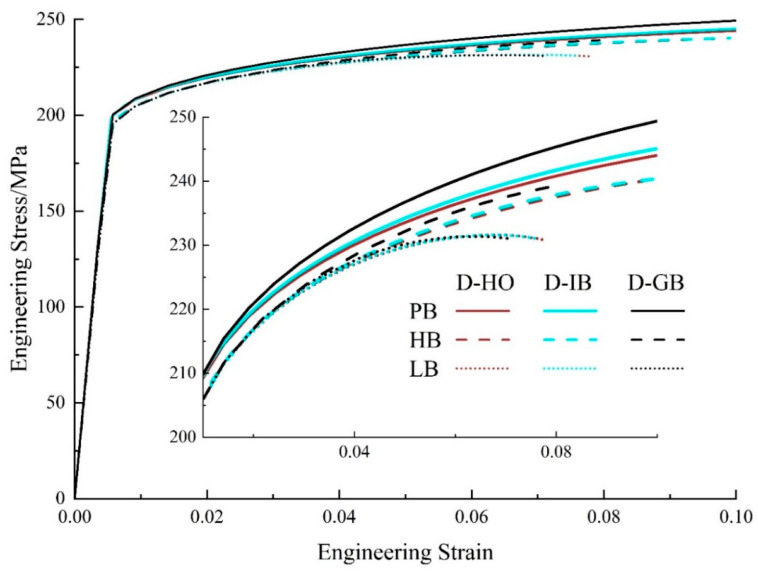
Stress–strain curves with different particle distributions and interface strengths.

Figure 10 presents the engineering stress–strain curves from the simulation for uniaxial tension with varied particle distributions and interface strengths. The inset is the enlarged image for the plastic flow part. The brown curves represent the distribution D-HO, the light blue curves are for the distribution D-IB, and the black curves are for the distribution D-GB. The solid, dashed, and dotted curves are for interfaces PB, HB, and LB, respectively. Several salient points can be noted in the figure, (1) When the interface property is the same (i.e., the same line style), the black curves show the highest flow stress compared to the other two colors. It indicates that when all the particles are located along the grain boundaries, it could generate the best hardening effect. (2) When the particle distribution is the same (i.e., the same color), the solid curves show the highest flow stress and the dotted curves show the lowest one. That is to say, when the interface changes to LB->HB->PB, the hardening effect will keep increasing. (3) No matter what color is, the solid curves show the highest flow stresses, while the three dotted curves give the lowest ones. This indicates that the influence of interface properties outweighs the effect of particle distribution on precipitation hardening. When the interface condition changes to PB->HB->LB, the influence of particle distribution on flow stress becomes almost negligible since the three dotted curves in Figure 10 almost coincide with each other. (4) It is obvious that among all the curves, the black solid line shows the highest flow stress. This means that when all the particles are located along grain boundaries and the interface between particles and the matrix is perfect, the effect of precipitation hardening reaches a maximum, and the resultant material exhibits the highest flow stress.

**Figure 11:**
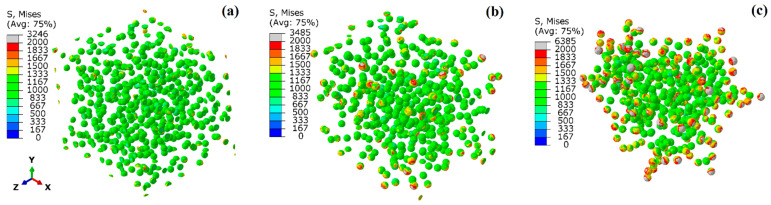
Stress status of SPPs with varied distributions of (**a**) homogeneous (D-HO), (**b**) intragranular boundary (D-IB), (**c**) grain boundary distribution (D-GB) at a strain of 10% with the PB interface.

**Figure 12:**
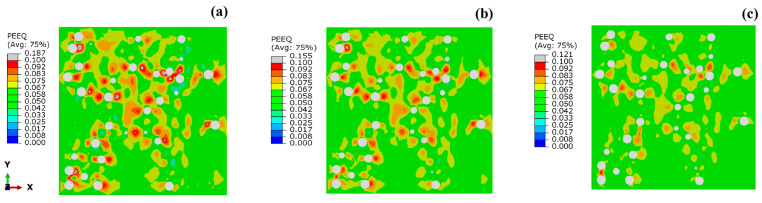
Equivalent plastic strain of the matrix with varied interface conditions: (**a**) PB, (**b**) HB, (**c**) LB at a strain of 7% with the D-GB distribution.

To further reveal the influence of different distributions and interface strengths on the hardening effect of SPPs, Figure 11 presents the contours of von Mises equivalent plastic stress in SPPs when the applied strain is 10% with the same PB interface. It can be seen that the stress distribution is more uniform on the SPPs with homogeneous distribution (see Figure 11a). The SPPs with the distribution D-GB exhibit higher stresses (see Figure 11c). This implies that, with the same interface properties, the SPPs all distributed at grain boundaries experience more pronounced stress concentration, resulting in a more effective transfer of loads to the particles in comparison to the other two distributions. Figure 12 shows the contours of the equivalent plastic strain in the matrix with the varied interface conditions when the applied strain is 7% (since the maximum strain occurs ~7% for the LB interface) with the same D-GB distribution. It is evident that the distribution of the equivalent plastic strain in the matrix is nonuniform, with higher values near the SPPs. When the interface condition changes to LB->HB->PB, the values of the equivalent strain in the matrix gradually increase. This means that a higher interface strength could result in a higher local stress concentration in the matrix.

To quantitatively analyze the load-bearing capability of particles, two indicators—the ratio of average stress and the average interface damage—are employed. The average stresses along the loading direction for particles and matrix are denoted *σ^p^* and *σ^m^*, respectively, and are obtained using a volumetric estimation method [30] as follows:

(6) $$\sigma^{p}=\frac{1}{V_{p}}\int \sigma_{11(p)}dV_{p}$$

(7) $$\sigma^{m}=\frac{1}{V_{m}}\int \sigma_{11(m)}dV_{m}$$

where $V_{p}$ and $V_{m}$ are the volume of particles and matrix, respectively.

The ratio of average stress (q) is defined as $q=\sigma^{p}/\sigma^{m}$, which evaluates the internal stress shared between the particles and matrix. A higher value of q indicates a greater load-bearing capacity undertaken by the particles.

The average interface damage *D* is evaluated by the area average method as follows [30]:

(8) $$D=\frac{1}{S}\int (SDEG)dS$$

where *S* is the interface area. *SDEG* is the scalar stiffness degradation, and it represents the overall damage in the cohesive element. It is initially 0. With damage initiation and its gradual evolution, *SDEG* increases from 0 up to 1. When *SDEG* reaches 1, the cohesive element will be removed to indicate interface debonding.

**Figure 13:**
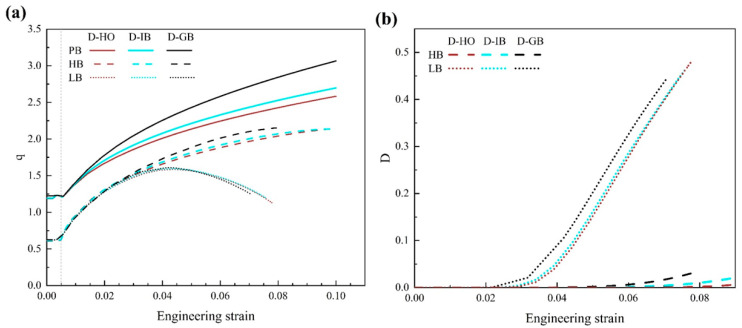
(**a**) The ratio of average stress and (**b**) the average interface damage of the Mg alloy with different particle distributions and interface strengths.

Figure 13a presents the ratio of average stress, and Figure 13b shows the average interface damage for the interfaces HB and LB since the perfect interface will not be damaged. In Figure 13a, for all interfaces, when the strain is less than 0.005, the ratio remains constant. This is the stage of elastic deformation. When the strain is larger than 0.005 and the plastic deformation proceeds, the ratio of average stress increases notably with strain. It means that particles will share more loads as the plastic strain increases. Consistent with the abovementioned analysis, the stronger the interface strength, the better the load-bearing capacity of particles is since all the solid curves show the highest ratio, followed by the dashed and the dotted curves showing the lowest ratio.

Another interesting point is worth noting that all three dotted curves show an obvious decrease when the strain is larger than ~4% in Figure 13a. In other words, for the interface with low strength (i.e., LB), the load-bearing capacity of particles is diminished as the strain increases. To check the damage of the LB interface, Figure 13b shows that at a strain of ~4%, the average interface damage of three dotted curves starts to rise steeply. Therefore, it is obvious that as the plastic deformation proceeds, the interface with low strength will result in a fast increase of interface damage and diminished hardening effect of particles at the microscale and corresponding strain softening of the material at the macroscale (as shown in Figure 10). This can even result in early failure and low ductility of the material, as indicated by the dotted curves in Figure 10.

**Figure 14:**
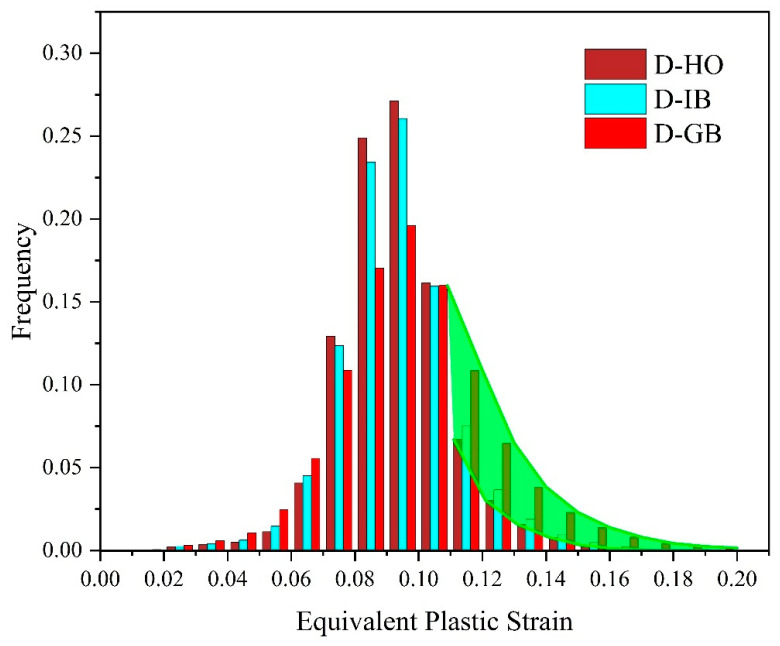
Histogram of an equivalent plastic strain of all the fine elements in the matrix (the green shadow highlights the equivalent plastic strain larger than 0.1).

Figure 14 presents a histogram of the equivalent plastic strain in the matrix when the applied strain is 10% for the PB interface. It is the statistical result of the equivalent plastic strain for all the fine elements of the matrix. The brown color is for the D-HO distribution, the light blue color is for D-IB, and the red color is for D-GB. As highlighted by the green shadow area in the figure, when the equivalent plastic strain is larger than 0.1, the frequency of the D-GB distribution (i.e., the red bar) is much higher than the other two distributions. This indicates that when all the particles are located along grain boundaries, it could result in more severe local plastic deformation around these particles than the other two distributions. Such severe local plastic deformation and corresponding high local stress concentration may result in early failure and low ductility of the materials if the interface is not perfect, as indicated by the black dashed and dotted curves in Figure 10.

### 4.2. Effect of Size Ratio of Grain to Particle and Interface Strength

**Figure 15:**
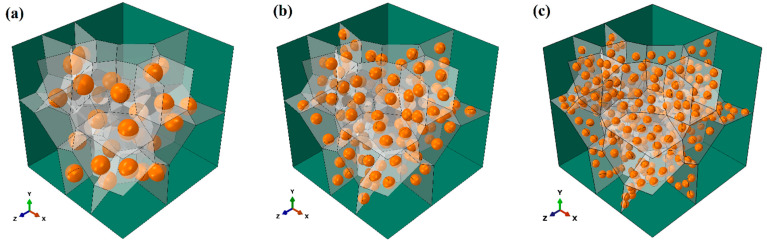
Geometrical models with varied ratios: (**a**) 3:1, referred to as Ratio 3, (**b**) 5:1, referred to as Ratio 5, (**c**) 7:1, referred to as Ratio 7 (gray planes denote the location of grain boundaries).

To assess the impact of the ratio of grain size to particle diameter on the mechanical properties of the Mg alloy with a fixed grain size, three diameters of spherical particles are adopted, and the ratios are 7:1, 5:1, and 3:1. Similar to the previous part, three interface strengths between the particles and matrix are considered. To concentrate on the two factors, all the models employ the D-GB particle distribution and the same volume fraction of 4% spherical particles. For convenience, the models with ratios of 7:1, 5:1, and 3:1 are denoted as Ratio 7, Ratio 5, and Ratio 3, respectively. The geometrical models with three size ratios are shown in Figure 15.

**Figure 16:**
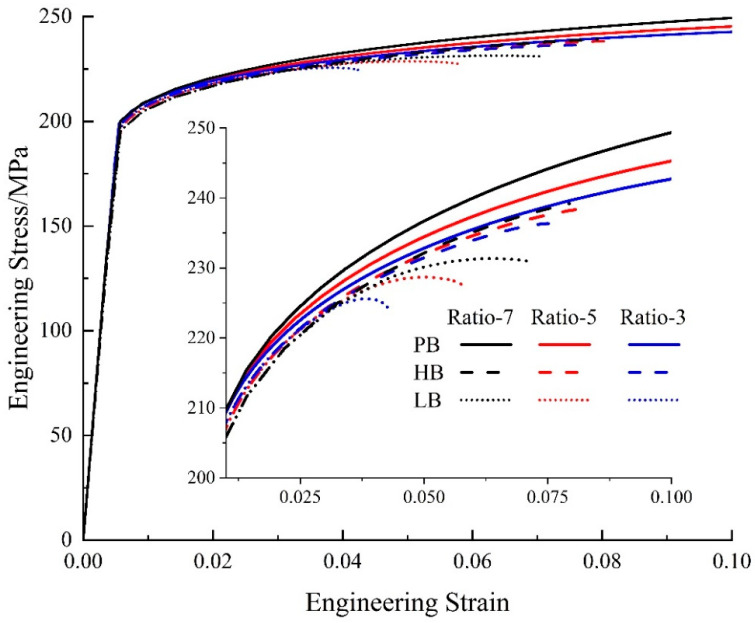
Stress–strain curves with different size ratios and interface strengths for particle distribution D-GB.

Figure 16 presents the engineering stress–strain curves from simulation for uniaxial tension with varied ratios and interface strengths. The inset is the enlarged image for the plastic flow part. The black curves represent Ratio 7, the red curves are for Ratio 5, and the blue curves are for Ratio 3. The solid, dashed, and dotted curves are for interfaces PB, HB, and LB, respectively. In Figure 16, two interesting points can be observed that. (1) When the size ratio is the same (i.e., the same color), the hardening effect of particles will keep increasing when the interface changes to LB->HB->PB. (2) Similar to the previous section, the solid curves show the highest flow stresses, while the three dotted curves present the lowest flow stresses. This indicates that the influence of interface properties is stronger than the effect of size ratio on precipitation hardening.

**Figure 17:**
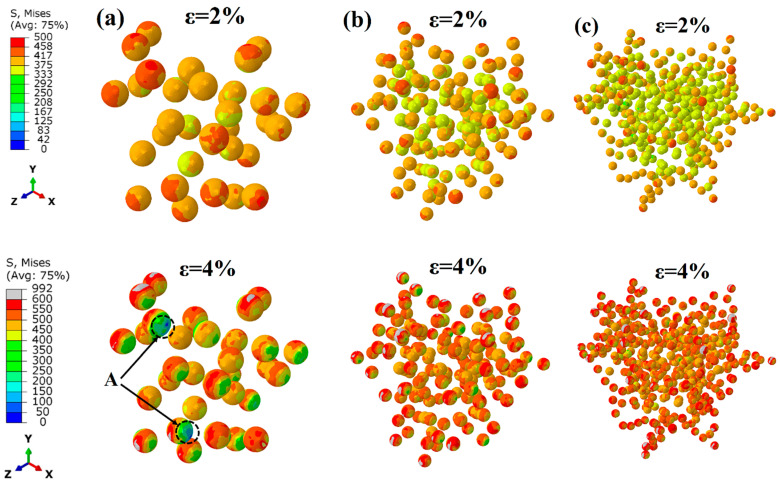
Stress status of SPPs with the LB interface and a varied size ratio of (**a**) 3:1, (**b**) 5:1, (**c**) 7:1 at applied strains of 2% and 4% for particle distribution D-GB.

To further reveal the influence of different size ratios and interface strengths on the hardening effect of SPPs, Figure 17a–c present the contours of von Mises equivalent plastic stresses (i.e., the deviatoric component of the stress state for plastic deformation [53]) in SPPs with different size ratios at applied strains of 2% and 4% for the LB interface. In the early stages of loading at a strain of 2%, the larger-size particles exhibit higher equivalent stresses since the red regions of SPPs are relatively more extensive in Figure 17a. However, in the later stages of loading at a strain of 4%, some particles with a size ratio of 3 change to blue color, as highlighted by the black ellipses in Figure 17a. This implies that the flow stress decreases in larger particles because interface damage occurs.

**Figure 18:**
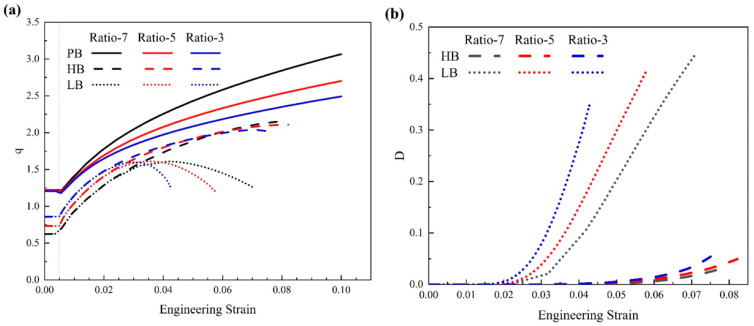
(**a**) The ratio of average stress and (**b**) the average interface damage of the Mg alloy with different size ratios and interface strengths for particle distribution D-GB.

Figure 18a presents the ratio of average stress, q, and Figure 18b shows the average interface damage for the HB and LB interfaces. One observation in Figure 18a is that for interfaces HB and LB, when the strain is less than ~3%, the ratio of average stress, q, keeps increasing from the black curves to the blue curves. That is to say, the larger particles could carry out more loads during deformation than the smaller particles with the same volume fraction. However, as the strain increases, the values of q reverse: the black curves are higher than the blue curves. Regarding the interface damage in Figure 17b, the reason can be found that for either the LB (i.e., the dotted curves) or HB (i.e., the dashed curves) interface, the damage evolves faster for the larger particles (i.e., the blue color) than the smaller particles (i.e., the black color). Such fast-developed interface damage could deteriorate the hardening effect of particles significantly. In addition, the interface damage develops much faster with the LB interface than with the HB interface. It could result in early failure and low ductility of the materials, as shown in Figure 15.

However, there is one opposite trend observed in Figure 18a for the perfect interface: the black curve shows the highest value of q. That is to say, the smaller particles can carry out more loads than the larger ones during deformation. Consistently, at the macroscale, the materials with smaller particles (i.e., the black solid curve) show the highest flow stress in Figure 15. This is an interesting point. To make smaller particles generate a stronger hardening effect, the interface between the particles and matrix must be strong enough to be the perfect interface applied in this work.

### 4.3. Effect of Particle Orientation and Interface Strength

**Figure 19:**
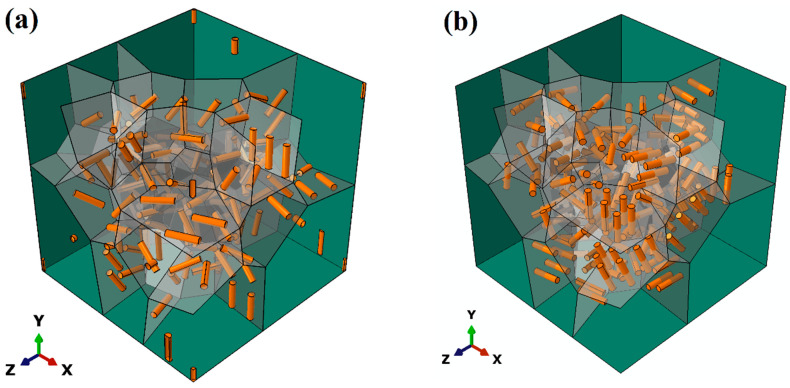
Cylindrical particle orientations (**a**) parallel to (denoted by Orien-0) and (**b**) perpendicular to (denoted by Orien-90) the grain boundaries (denoted by gray planes).

The orientation of the second-phase particles within the matrix could affect the mechanical properties of the Mg alloy significantly. In this part, particles are modeled as small cylinders with an aspect ratio of 4.6, and the volume fraction is 1.32%. All the parameters of the particles are obtained from experiments and listed in Table 2 in Section 3.4. Cylindrical particles are all distributed along the grain boundaries. Figure 19a shows that all the small cylinders are located parallel to the grain boundaries, denoted by Orien-0, while the cylinders are located perpendicular to the grain boundaries in Figure 19b, denoted by Orien-90. Three interface properties are applied for the two arrangements of particle orientation, respectively.

**Figure 20:**
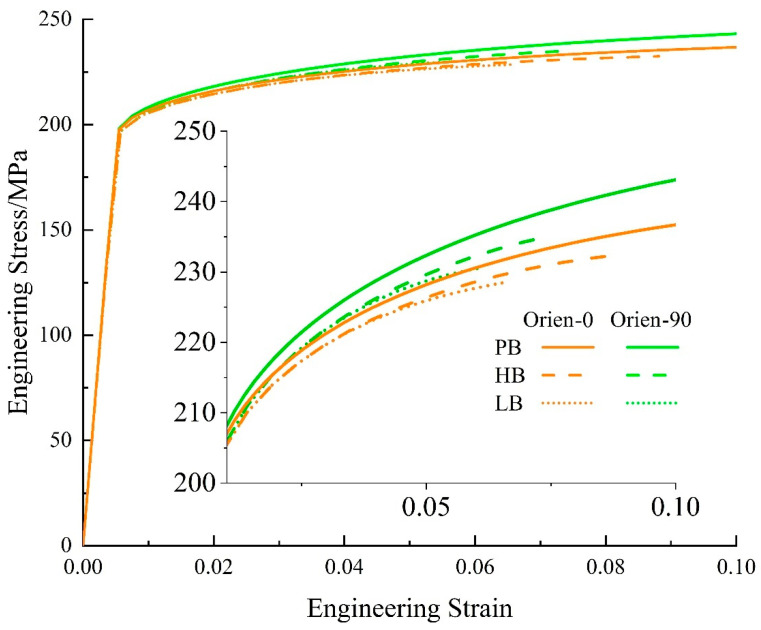
Stress–strain curves with particle orientations of Orien-0 and Orien-90 and varied interface strengths.

Figure 20 presents the engineering stress–strain curves from the simulation for uniaxial tension with varied particle orientations and interface strengths. The inset is the enlarged image for the plastic flow part. The brown curves represent Orien-0 and the green curves are for Orien-90. The solid, dashed, and dotted curves are for interfaces PB, HB, and LB, respectively. In Figure 20, it is evident that all the green curves show higher flow stress than that from the brown curves. This means that the influence of particle orientation on precipitation hardening outweighs the effect of interface strength. The perpendicular orientation (i.e., green curves) exhibits a stronger hardening effect than that from parallel orientation (i.e., brown curves). In addition, for the same orientation (i.e., the same color in Figure 20), the PB interface could generate the best hardening effect. However, the difference between interfaces HB and LB is negligible, as shown by the dashed and dotted curves.

**Figure 21:**
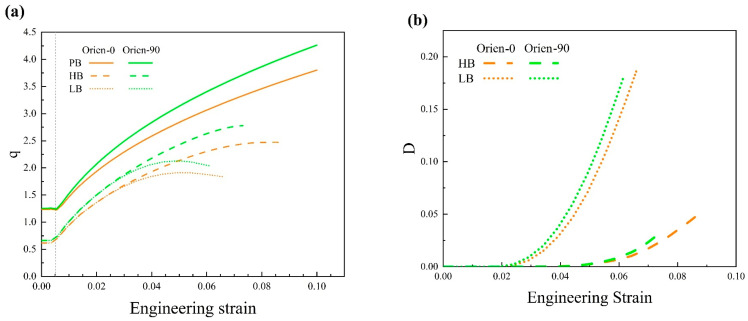
(**a**) The ratio of average stress and (**b**) the average interface damage of the Mg alloy with different particle orientations and interface strengths.

In Figure 21a, consistent with the above analysis, for interfaces HB and LB with the same orientation (i.e., the same color), the ratios of average stress, q, almost coincide with each other, which indicates the same load-bearing capacity. Similar to the results in Section 4.1 and Section 4.2, the LB interface always results in a steep increase in interface damage, as shown by the dotted curves in Figure 21b. This is also reflected by the corresponding low ductility of materials at the macroscale in Figure 20. Another point in Figure 21b that is worth noting is that for the same interface, the green curves increase faster than the brown curves. That is to say, the perpendicular orientation could generate more damage than that from parallel orientation as the deformation proceeds, although it could generate a stronger hardening effect.

**Figure 22:**
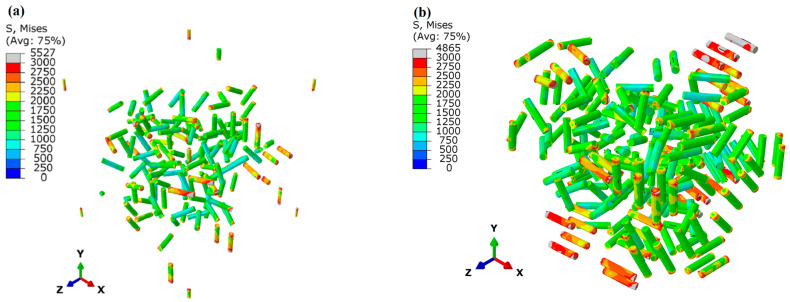
Stress status of SPPs with varied orientations: (**a**) Orient-0 and (**b**) Orient-90 at a strain of 10% with the PB interface.

Figure 22 shows the stress status of the cylindrical particles in the models Orien-0 and Orien-90 at a strain of 10% with the same PB interface. It is evident that the stress level in the small cylinders for the orientation Orien-90 (see Figure 22b) is generally higher than that for the orientation Orien-0 (see Figure 22a). This is consistent with the result in Figure 21a, which shows that the solid green curve has a higher value of the ratio q than that from the solid brown curve, which means a higher load-bearing capacity for the particles in the orientation Orien-90.

## 5. Conclusions

This work investigates the influence of second-phase particles on the mechanical properties of Mg alloys with the rare earth element Gd. Three-dimensional FE models with the explicit incorporation of second-phase particles are established and calibrated with the experimental results of the alloy Mg-1Gd. The influences of particle-related factors, such as distribution, size, and orientation, are investigated in detail, coupled with varied interface properties between particles and the matrix. Several conclusions can be drawn.

1. The influence of interface strength outweighs the effect of particle distribution in terms of precipitation hardening. The higher interface strength could cause higher plastic flow stress. When all the second-phase particles are located at grain boundaries, they could generate the best hardening effect compared to the homogeneous distributions (D-HO) and intragranular boundary distributions (D-IB).
2. The influence of interface strength exceeds the effect of particle size on precipitation hardening. Larger particles in the matrix show stronger load-bearing capacity, while they also bring high local stress concentration, which may result in low ductility. However, when the interface is strong enough (i.e., perfect interface), the smaller particles could generate a higher hardening effect.
3. The influence of the orientation of cylindrical particles at grain boundaries outweighs the effect of interface strength on precipitation hardening. The perpendicular orientation with respect to grain boundaries exhibits a stronger hardening effect than that from parallel orientation.

## Figures and Tables

**Table 1:** Parameters for the interface.

Property	HB	LB
Interface stiffness (GPa)	100	100
Interface strength (MPa)	500	300
Fracture energy (J/m^2^)	5	3
Failure displacement (μm)	0.02	0.02

**Table 2:** Parameters for the Gd-rich second-phase particle.

	Maximum Feret Diameter (μm)	Volume Fraction of Particle (%)	Aspect Ratio
SPP	1.38	1.32	4.60

**Table 3:** The mechanical properties of the alloy Mg-1Gd.

Material	Young’s Modulus, E (GPa)	Poisson’s Ratio, υ	Plastic Property, σ
α-Mg	37.57	0.3	195 + 180 ε^0.5^
SPP [33]	57.8 ± 4.79	0.25	-

## Data Availability

Data are contained within the article.
